# An unusual case of hyalohyphomycosis due to *Purpureocillium lilacinum* in a patient with myasthenia gravis

**DOI:** 10.18502/cmm.4.2.62

**Published:** 2018-06

**Authors:** Ramya Raghavan, Gomathi Chithra, Sanal Fernandez, Bettadpura Shamana Suryanarayana, Rakesh Singh

**Affiliations:** 1 Department of Microbiology, Jawaharlal Institute of Postgraduate Medical Education and Research, Puducherry, India; 2 Department of Medicine, Jawaharlal Institute of Postgraduate Medical Education and Research, Puducherry, India

**Keywords:** Hyalohyphomycosis, Immunocompromised patient, Myasthenia gravis, Paecilomyces lilacinus, Purpureocillium lilacinum

## Abstract

**Background and Purpose:**

*Purpureocillium lilacinum* (previously known as *Paecilomyces lilacinus*) and* Paecilomyces variotii* cause hyalohyphomycosis.

**Case report:**

In this study, we present a case of multiple subcutaneous abscesses of the lower limbs due to *Purpureocillium lilacinum *in a patient with myasthenia gravis and uncontrolled diabetes. Subcutaneous involvement of the lower limbs with this fungus is an unusual presentation. Pus aspirate collected on multiple occasions revealed hyaline septate hyphae under microscopic examination and *Purpureocillium lilacinum* grew on Sabouraud Dextrose Agar*. *The patient was initially treated by surgical excision and itraconazole therapy. Swelling regressed but discharge was noticed from the excision site after three months of itraconazole therapy. Culture from the discharge material yielded the same fungal growth. Treatment was changed to ketoconazole and he responded.

**Conclusion:**

This case report emphasizes the importance of identifying *Purpureocillium lilacinum *at an unusual site like the lower limbs in an immunocompromised patient. Ketoconazole may be used as an alternative treatment option for hyalohyphomycosis caused by *Purpureocillium lilacinum*.

## Introduction

*Purpureocillium lilacinum* (earlier known as *Paecilomyces lilacinus*) and* Paecilomyces variotii* cause hyalohyphomycosis. Other species occasionally associated with the disease are *Paecilomyces* *marquandii *and* Paecilomyces javanicus *[1]. *Purpureocillium lilacinum* is a saprophytic fungus with ubiquitous distribution [2]. It causes oculomycosis or skin and soft tissue infections and sometimes deep-seated infection [2]. 

*Purpureocillium lilacinum* is reported to be resistant to the most commonly used antifungals like fluconazole, itraconazole, and terbinafine. Voriconazole and posaconazole are effective antifungals used for treating this infection. Species identification of the causative agent is crucial as *Paecilomyces variotii* is resistant to voriconazole [3]. Ketoconazole is rarely used in the treatment of hyalohyphomycosis caused by *Purpureocillium lilacinum*. Wessolossky et al. treated a patient of olecranon bursitis caused by *Purpureocillium lilacinum* by ketoconazole and surgical debridement [4]. Herein, we described a case of subcutaneous soft tissue abscess of the lower limbs caused by *Purpureocillium lilacinum* in an immunocompromised patient treated with ketoconazole.

## Case report

A 34-year-old male driver who was suffering from myasthenia gravis presented to the medical emergency department of **Jawaharlal Institute of Postgraduate Medical Education and Research**, Puducherry, India, with increasing fatigability, ptosis, and diplopia for seven days. The patient was a native from the same city. Informed consent was obtained from the patient for using his clinical information and images for the academic and research purposes.

**Figure 1:**
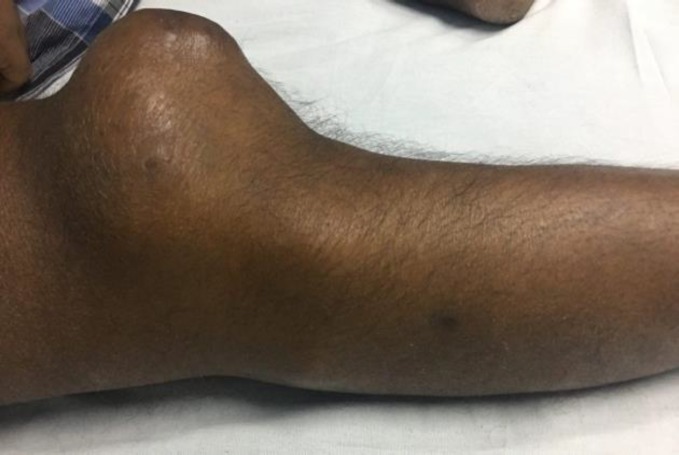
Clinical image of the patient showing knee swelling

The patient had undergone thymectomy four years ago for myasthenia gravis and was on tablet prednisolone 40 mg once daily, tablet pyridostigmine 90 mg six-hourly for five years, and tablet cyclosporine 200 mg twice daily for the last one year. The patient had also developed steroid-induced diabetes mellitus one year ago and had received insulin therapy. His vitals were stable. There were multiple painless swellings in the right leg region for the preceding one month; the biggest swelling measured 10 × 7 cm near the knee region (Figure 1) and multiple small swellings were noticed between the knee and ankle. There was no history of previous similar swellings. His random blood sugar at the time of admission was 345 mg/dl and HbA1c 10.7%. Insulin dose was adjusted and it reduced to 135 mg/ml on the fifth day of admission. 

**Figure 2:**
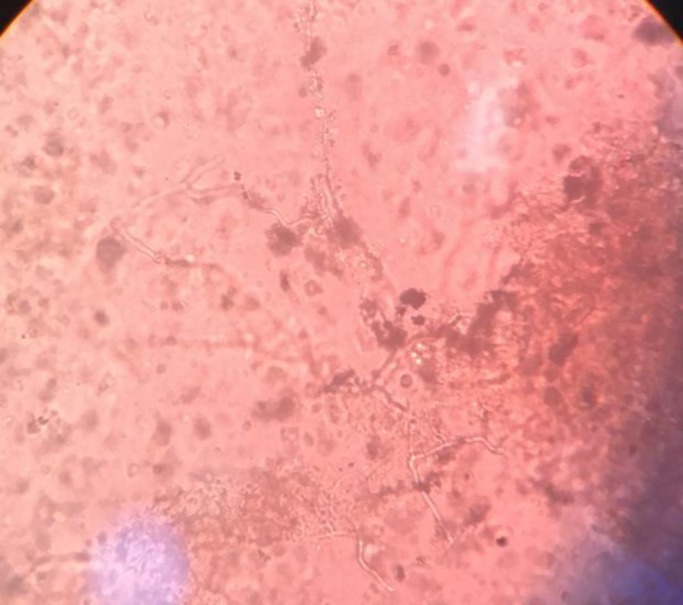
10% potassium hydroxide mount showing thin, septate, hyaline and branched hyphae (400X magnification)

**Figure 3:**
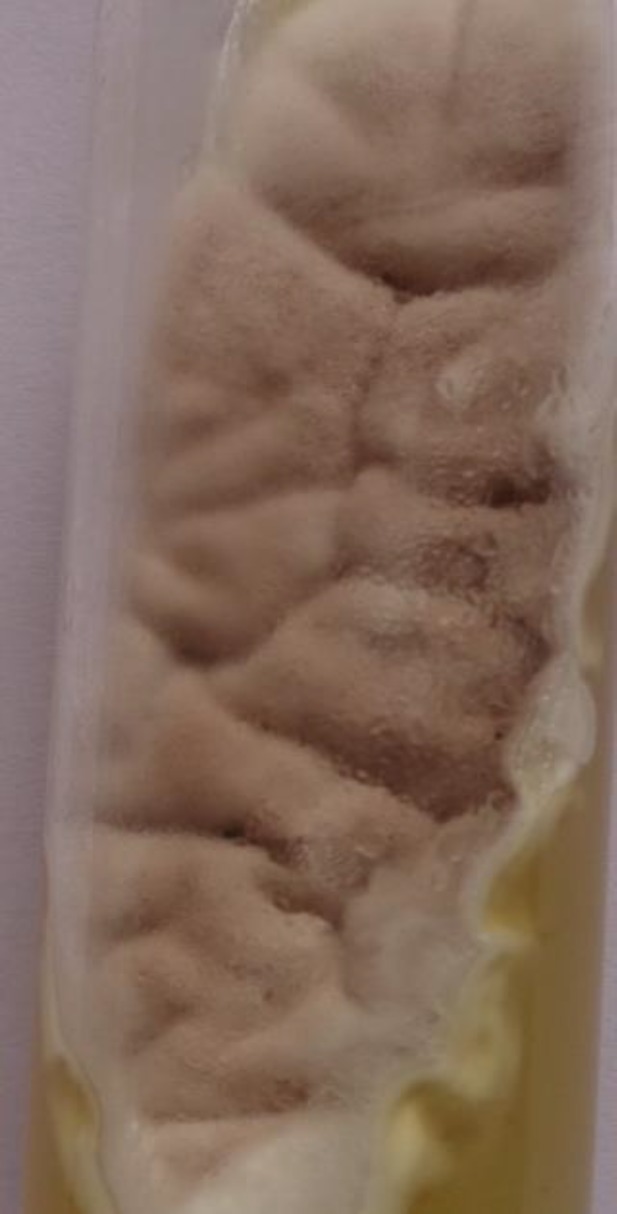
Culture on Sabouraud Dextrose Agar (SDA) showing flat and velvety lilac colour colonies

**Figure 4:**
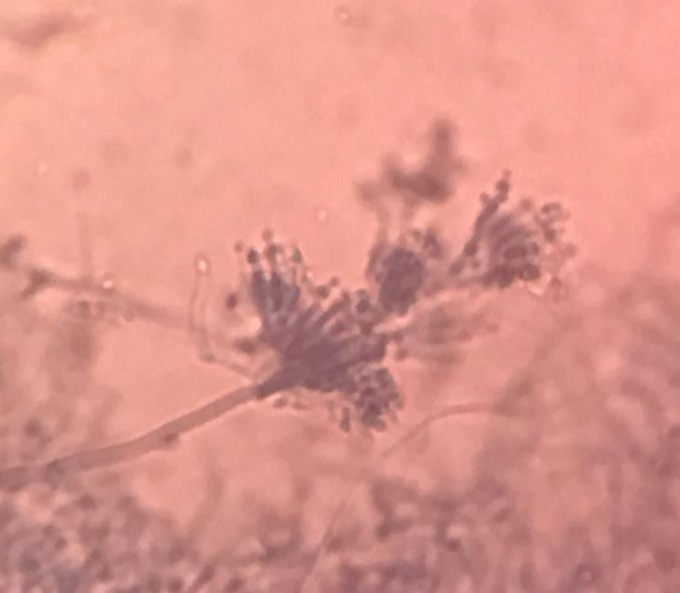
Lactophenol cotton blue showing thin hyaline septate hyphae with irregularly branching conidiophores, elongated phialides, which have a tapering end and are densely clustered (400X magnification)

Ultrasound of the swelling near the right knee showed multiloculated collections with moving echoes in the knee joint. The pus was yellowish, not foul smelling and not blood stained. The 10% potassium hydroxide (KOH) mount showed thin, septate, hyaline, and branched hyphae (Figure 2). The pus was inoculated on Sabouraud Dextrose Agar (SDA) and incubated at 25°C in multiple tubes. All the culture tubes grew flat, densely floccose, and velvety lilac colour colonies and the reverse was off-white within seven days of incubation (Figure 3). Lactophenol cotton blue stain (LPCB) preparation of the growth revealed thin hyaline septate hyphae with irregularly branching conidiophores. Phialides were more elongated and densely clustered and had a tapering end. The conidia were elliptical, in long unbranched chains, and phenotypically identified as *Purpureocillium lilacinum* (Figure 4).

*Purpureocillium lilacinum* was again isolated from two more pus aspirates, which were sent at varied intervals over the next one week. Antifungal susceptibility testing could not be performed because of logistic reasons. The fungal culture was confirmed by *National Culture Collection of Pathogenic Fungi****, ***Centre of Advance Research in Medical Mycology, WHO Collaborating Centre, Chandigarh, India (ID no*.* IL2892) by sequencing of internal transcribed spacer (ITS) region. The sequence was submitted to the gene bank database with the accession number MH714911. 

The patient was started on antifungal tablet itraconazole 300 mg once daily. The swellings were surgically excised and histopathological examination of the excised tissue revealed thin septate hyaline hyphae. After three months of itraconazole therapy, the patient had no new swellings but complained of a serous discharge at one of the excision sites. The discharge was collected and 10% KOH mount of the discharge was negative for fungal elements, but the culture grew *Purpureocillium lilacinum* again. Itraconazole therapy was extended for another month at the same dose, but the patient had recurrence of small swellings. Treatment was changed to tablet ketoconazole 200 mg once a day for three months. The swellings responded well to ketoconazole. He remained asymptomatic and swelling regressed after one month of ketoconazole therapy. 

## Discussion

*Purpureocillium lilacinum* is one of the emerging fungal pathogens [2]. The incidence of hyalohy-phomycosis due to this organism is on the rise and is associated with a mortality of around 25% [4]. It causes sinusitis, keratitis, wound infections, as well as skin and nail infections like onychomycosis, vaginitis, and endophthalmitis [5-7]. Deep infections are rare, though a case of osteomyelitis [2] and a case of disseminated disease [7] have been reported. Though toe nail onychomycosis has been described previously [6], subcutaneous involvement of the lower limbs has not been reported to the best of our knowledge. Hence, the present case is an unusual presentation of *Purpureocillium lilacinum*. This patient was having two recognized risk factors for opportunistic infections, namely long-term immunosuppresive therapy and poorly controlled diabetes mellitus.

The mode of entry of the fungus usually involves traumatic inoculation, inhalation, and rarely through indwelling catheters [8] and fluids contaminated with conidia of *Purpureocillium lilacinum* [8, 9]. This organism has been found to be useful as a bio-pesticide to control nematodes that attack plant roots [10]. There was no known exposure to such bio-pesticides for the patient. He might have acquired the fungi through traumatic inoculation of the conidia, but the exact source is highly uncertain in this patient. 

Identification of *Purpureocillium lilacinum* was made by its lilac obverse colour, and penicillus type of culture morphology, which differentiated it from *Penicillium* spp. and *Paecilomyces* spp. The ability to sporulate in tissue is one of the characteristic features of this fungus, which may be used for its identification in biopsy specimen [11]. Molecular techniques such as ribosomal DNA sequencing may be utilized to confirm its identity [12]. 

It is essential to differentiate *Purpureocillium lilacinum *and* Paecilomyces variotii* as causative agents of hyalohyphomycosis because *Purpureocillium lilacinum* responds poorly to itraconazole, amphotericin B, and echinocandins but responds well to voriconazole [13]. In contrast, *Paecilomyces variotii *is susceptible to most antifungal drugs, except voriconazole. This patient did not respond to itraconazole therapy and voriconazole could not be started due to economic constraints. Ketoconazole was tried and the patient responded. This finding is in accordance with the case reports by Sharma et al. [14] and Wessolossky et al. [4]. The present case is the third case who responded to ketoconazole. Accordingly, ketoconazole may be used as an alternative treatment for *Purpureocillium* *lilacinum* infections in resource-limited settings. More studies are required to establish this observation.

## Conclusion

This case highlights the possibility of hyalohy-phomycosis due to *Purpureocillium lilacinum *at an unusual site like the lower limbs. In resource-limited settings, ketoconazole may be used as an alternative treatment option. 
